# Artificial intelligence based fusion imaging streamlining mitral transcatheter edge-to-edge repair

**DOI:** 10.1093/ehjimp/qyag048

**Published:** 2026-03-12

**Authors:** Patric Biaggi, Roberto Corti, Oliver Gaemperli, Peter Wenaweser, Nicolas Brugger, Fabien Praz, Leo Timmers, Martin Swaans, Susheel K Kodali, Rebecca T Hahn

**Affiliations:** Heart Clinic Hirslanden, Witellikerstrasse 40, Zurich 8032, Switzerland; University of Zurich, Zurich, Switzerland; Heart Clinic Hirslanden, Witellikerstrasse 40, Zurich 8032, Switzerland; University of Zurich, Zurich, Switzerland; Heart Clinic Hirslanden, Witellikerstrasse 40, Zurich 8032, Switzerland; University of Zurich, Zurich, Switzerland; Heart Clinic Hirslanden, Witellikerstrasse 40, Zurich 8032, Switzerland; Department of Cardiology, University Hospital, University of Bern, Bern, Switzerland; Department of Cardiology, University Hospital, University of Bern, Bern, Switzerland; Department of Cardiology, University Hospital, University of Bern, Bern, Switzerland; St Antonius Hospital, Nieuwegein, The Netherlands; St Antonius Hospital, Nieuwegein, The Netherlands; Department of Medicine, Columbia University Irving Medical Center, New York, NY, USA; Department of Medicine, Columbia University Irving Medical Center, New York, NY, USA

**Keywords:** interventional echocardiography, mitral edge-to-edge repair, artificial intelligence, fusion imaging

## Abstract

**Aims:**

Precise imaging is critical for procedural success in mitral transcatheter edge-to-edge repair (M-TEER), yet conventional fluoroscopy and echocardiography may lead to miscommunication and suboptimal device placement. The aim was to test the clinical utility of DeviceGuide EchoNavigator SmartVue (Philips Healthcare), a novel artificial intelligence-based fusion imaging software that automatically tracks the PASCAL Ace device and aligns live 3D transoesophageal echocardiography with fluoroscopy.

**Methods and results:**

In this prospective multi-centre study, DeviceGuide was evaluated in four structural heart centres in the USA, The Netherlands, and Switzerland in consecutive patients undergoing M-TEER with the PASCAL Ace device. Dedicated imaging modes support the procedure: target mode with trajectory overlays for real-time navigation, and a device mode that delivers continuous, auto-centred and auto-aligned device visualization throughout leaflet capture and closure. Interventional teams completed a structured qualitative questionnaire focusing on workflow, team discussions on optimal trajectory, perceived image quality and stability, and overall usefulness of the software during key procedural stages. Among 51 DeviceGuide-assisted M-TEER procedures, clinical teams rated the software as helpful or very helpful in guiding the intervention in most cases and reported improved discussion of optimal strategy. Main perceived advantages over conventional imaging were enhanced awareness and real-time feedback on trajectory and automated 3D-TEE views with continuous, auto-aligned device imaging during implantation.

**Conclusion:**

This AI-based fusion imaging approach demonstrated high perceived utility during M-TEER with PASCAL Ace and appeared to streamline workflow and team communication, supporting further studies to determine its impact on clinical outcomes.

## Graphical Abstract

**Graphical Abstract:**
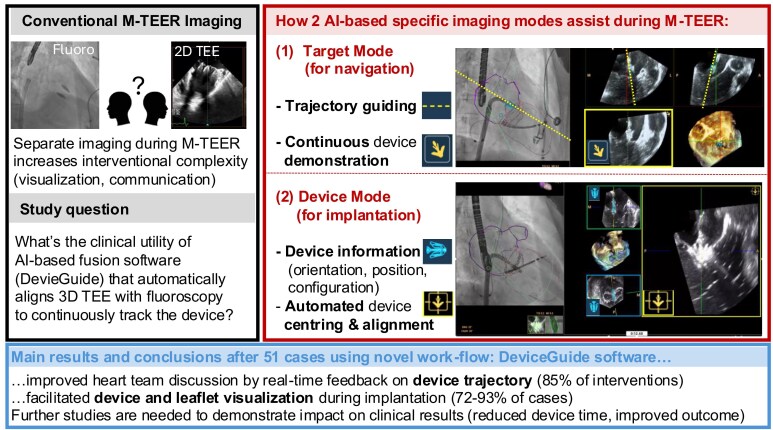

## Introduction

Mitral transcatheter edge-to-edge repair (M-TEER) is highly dependent on precise interventional imaging for procedural success. Operators must integrate information from X-ray fluoroscopy and three-dimensional transoesophageal echocardiography (3D-TEE) in real time to safely and precisely manipulate devices. This separation of imaging reference frames—combined with the dynamic nature of beating heart interventions—increases the risk of miscommunication, potentially prolongs procedures (up to 104 min in 2023^1^) and can limit the precision of device placement, especially in anatomically challenging cases. DeviceGuide (EchoNavigator SmartVue Investigational Device, Philips Healthcare, NL) is a novel artificial intelligence (AI)-based fusion imaging software developed to overcome these limitations. Using advanced deep learning algorithms, DeviceGuide can automatically recognize both the shape and orientation of the TEE probe and the M-TEER device (currently limited to PASCAL Ace, Edwards Lifesciences, USA) on fluoroscopic imaging and automatically transforms 3D-TEE data to continuously image the device and its location relative to the anatomy. Compared with earlier workflows that largely rely on manual view selection and adjustment, the investigated AI-based workflow adds device-aware view automation, enabling automatically generated and maintained device-augmented and device-centred views while reducing reliance on manual adjustments. The functionality of the investigational (prototype) software used in this study has been commercialized in EchoNavigator R5 product.

Here, we describe in detail the novel workflow using DeviceGuide and report the utility on the initial 51 patients. All patients consented to participate in the study reviewed by the respective ethics committees of the four participating centres (Heart Clinic Zurich, Switzerland; Department of Cardiology, University Hospital of Bern, Switzerland, Department of Cardiology, St Antonius Hospital, Nieuwegein, The Netherlands; and Department of Medicine, Columbia University Irving Medical Centre, New York, NY, USA). Immediately after the interventions, representatives of the interventional teams of Bern (imager), Zurich (imager), and New York (imager and interventionalist) gave structured qualitative feedback on paper-based case report forms regarding the impact of the software on the intervention. The counts of the NY team were added up and then divided by two in order to get only one response per site. The feedback covered the following aspects: accuracy of device detection to guide intervention, image quality of the novel device view in both modes during various stages of the procedure (device steering, leaflet grasp, and device closure), and impact on heart team discussion regarding strategy and trajectory to target. Most questions included five answer possibilities (i.e. not helpful at all, somewhat helpful, neutral, helpful, and very helpful), whereas questions regarding impact on heart team discussion were answered by yes or no.

### Three distinct imaging modes for specific procedural steps

**Figure 1:**
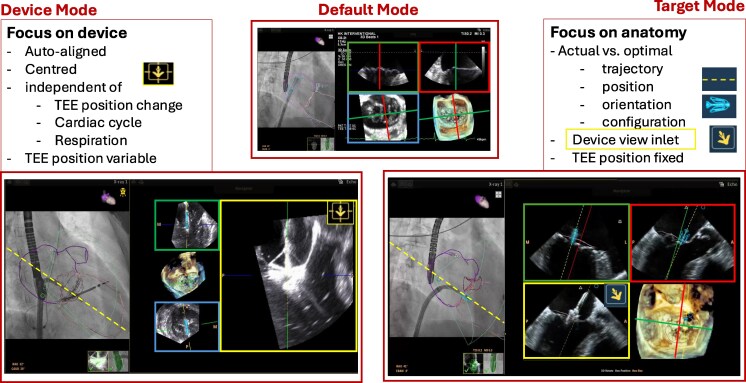
DeviceGuideConcept with 3 distinct imaging modalities. ‘Default mode’, comparable to standard 3D-TEE MPR is used to define the target lesion (crossing of red and green planes). ‘Target mode’ provides the actual trajectory path of the device (yellow dashed line) relative to the optimal trajectory (red and green lines) and continuously shows the device long-axis orientation relative to the surrounding anatomy (yellow plane). Device Mode maintains continuous visualization of the device, auto-aligning the long-axis imaging plane at the image centre throughout closure.

The procedural workflow is enhanced through a sequential use of three intuitive modes, each designed to optimize critical steps of mitral valve intervention (*Figure 1*). The ‘default mode’ [comparable to standard 3D-TEE multi-planar reconstruction (MPR) view] facilitates multi-disciplinary heart team discussion by allowing precise assessment of the mitral anatomy and defining the intervention target. ‘Target mode’ allows real-time device navigation by standard red and green MPR planes demonstrating the defined target. In addition, it provides the actual trajectory path of the device (yellow dashed line) and continuously shows the actual device long-axis orientation relative to the surrounding anatomy (yellow plane). The device is further enhanced by a blue icon real-time overlay. ‘Device mode’ used for device implantation maintains continuous, auto-centred visualization of the device, auto-aligning the optimal imaging planes to keep the device at the imaging centre throughout closure.

### Facilitated device navigation by real-time feedback on trajectory

**Figure 2:**
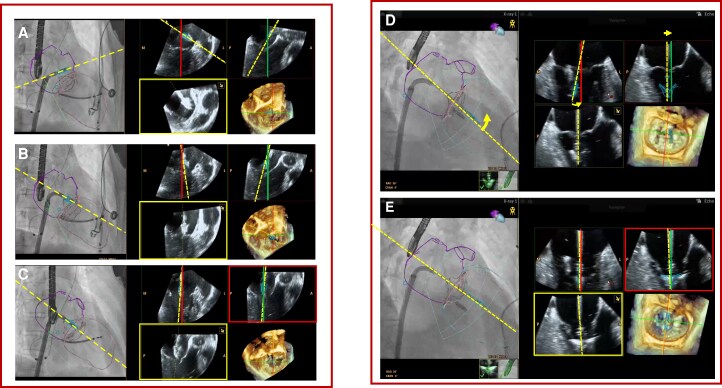
Target mode for navigation above and below the mitral valve. ‘Target mode’ integrates standard 3D MPR views with a new concept resembling an aircraft’s artificial horizon, using a yellow trajectory line to provide real-time feedback during device navigation towards the mitral target zone (Panel *A–C*; Supplementary data online, *Video S1*). Unlike the fixed green and red planes showing the ‘optimal’ trajectory, the yellow line and plane display the ‘actual’ device trajectory and orientation, enabling instant feedback and corrective adjustment (Panel *D* and *E*; Supplementary data online, *Video S2*).

Target mode (*Figure 2*) combines standard 3D MPR views with a novel imaging concept similar to the concept of the artificial horizon in airplanes. The yellow trajectory line (both on TEE and on fluoroscopy) aims to guide the operator from entry into the left atrium to the optimal mitral target zone (Panels *A–C*; Supplementary data online, *Video S1*). In contrast to the green and red standard 3D MPR views locked to the target zone and therefore indicating the ‘optimal’ target trajectory, the dashed yellow line represents the ‘actual’ trajectory. This instant feedback assists the heart team in making rapid corrections if the device deviates from the optimal line, thus potentially improving targeting accuracy and reducing the risk of suboptimal device positioning. A second novelty is the yellow plane substituting the typical blue plane of standard 3D MPR. This plane autonomously and continuously images the device in its long-axis orientation relative to the surrounding anatomy. This is particularly helpful as the green and red planes are locked and thus do not necessarily show the device at all times. Once the yellow and red planes become identical, the correct device location and orientation at the target implant site is reached (Panel *C*). After crossing the mitral valve, ‘target mode’ further assist the interventional team by demonstrating both optimal and actual trajectory and device orientation without the need of image manipulation even at times of suboptimal image quality. Panels *D* and *E* (see Supplementary data online, *Video S2*) demonstrate how much cardiorespiratory motion can deviate the device from the optimal trajectory, and how the yellow dashed line facilitates the device steering corrections.

### Improved device visualization during implantation

**Figure 3:**
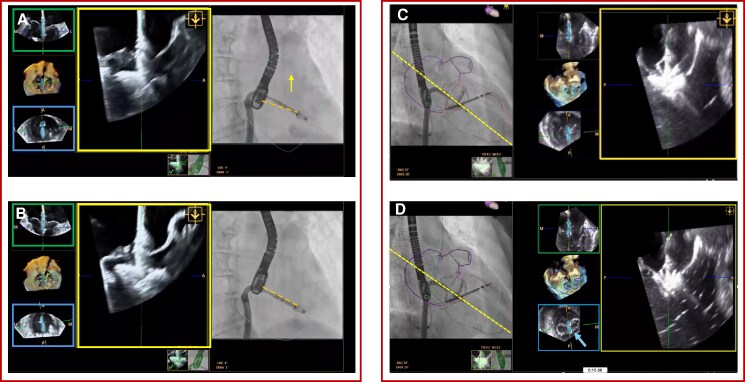
Device mode for overcoming shadowing and for device closure. Device mode provides a continuously centred and automatically aligned view of the implant, maintaining the device’s long-axis orientation despite patient or probe movement. This ensures uninterrupted visualization during TEE probe movements to overcome shadowing of the anterior mitral leaflet (Panel *A* and *B*; Supplementary data online, *Video S3*) or during closure (Panel *C* and *D*; Supplementary data online, *Video S4*), allowing precise and controlled leaflet capture with both device arms clearly displayed.

Once the implant position below the mitral valve has been reached, ‘device mode’ provides a focused device view continuously centring of and automatically aligning with the device during critical phases (*Figure 3*). In this mode, the software autonomously and dynamically reorients the multi-planar 3D-TEE reconstruction to maintain the long-axis view of the device at the imaging centre, regardless of changes in heart rhythm, patient respiration, or probe movements. In ‘device mode’, the red plane is interchanged with the blue plane always levelling at the centre of the device. Even when the probe position must be adjusted, for example to overcome acoustic shadowing (Panel *A* and *B*; Supplementary data online, *Video S3*), DeviceGuide updates the imaging planes automatically, maintaining continuous visualization without the need for manual plane readjustment. Furthermore, ‘device mode’ facilitates the imagers intent to optimally visualize the anterior and posterior arm of the device during device closure where precise and controlled leaflet capture is essential (Panel *C* and *D*; Supplementary data online, *Video S4*). In addition, the blue plane continuously demonstrates the device centre and thus gives immediate feedback on the amount of tissue bridge reached by the grasp (blue arrow, Panel *D*). Transgastric imaging, typically used to demonstrate the tissue gap but also known to be associated with greater risk of gastric bleeding complications^2^ can thus be avoided.

### Qualitative feedback on utility

For the first 51 patients treated using DeviceGuide software, the teams reported the software to be ‘helpful or very helpful’ in guiding the M-TEER intervention in 68.7% of cases. Ratings of ‘not helpful’ (4%) were mainly due to inadequate device recognition in the presence of multiple additional heart devices. According to the qualitative team feedback the software improved heart team discussion on optimal trajectory in 85.2% of interventions. Image quality was rated sufficient or highly sufficient during device steering, leaflet capture and device closure in 93.1%, 72.6% and 74.5%, respectively. The live display of the actual vs. intended trajectory lines was felt to be particularly beneficial to achieve a good technical interventional result as it enhanced implantation of the device perpendicular to the coaptation line.

## Conclusion

The results of this study are limited by the small number of patients and the mainly qualitative rather than quantitative results. Furthermore, the non-comparative nature of the study prevents a conclusive statement regarding the software’s ability to improve the technical or clinical outcome. Nevertheless, this novel AI-based fusion imaging concept showed high utility and appeared to support the workflow of the studied intervention teams. Reported key advantages were the enhanced strategy discussion of the intervention teams, supported by real-time feedback on trajectory and by continuous, auto-aligned imaging of the device during implantation. This software harbours the potential to improve procedural efficacy by enhancing device visualization, streamlining guidance, and thus reducing procedure time even for very experienced teams.^3^ AI-based technology is of increasing importance in view of the challenges interventional teams face during device guidance in complex anatomies.^4^ Further controlled studies are warranted to prove better technical outcomes when using DeviceGuide imaging during structural heart interventions compared with standard imaging.

## Supplementary Material

qyag048_Supplementary_Data

## Data Availability

The datasets generated and analysed during the current study are not publicly available due to ethical and legal restrictions. Participant consent and the research protocol approved by the institutional ethics committee did not include permission for the public sharing of raw patient data. Furthermore, the fusion software algorithm used in this study is the proprietary property of Philips Healthcare, and its underlying code is subject to commercial confidentiality agreements that prohibit public disclosure. Inquiries regarding the data supporting the findings of this study may be directed to the corresponding author, although access is subject to approval by the study sponsors and the ethics committee.

## Lead Author Biography

Dr Biaggi graduated from Medical School in Bern, Switzerland, with a doctoral degree in 1998. Swiss Board Certification in General Internal Medicine (2005) and Cardiology (2010). He completed a 2-year fellowship in echocardiography at Toronto General Hospital, Canada, specializing in 3D echocardiography and valvular heart disease. Habilitation at the University of Zurich, Switzerland, in 2013 and appointed Professor of Cardiology in 2023. Since 2013, serves as Head of Heart Imaging at Heart Clinic Zurich, with expertise in diagnostic and interventional echocardiography for valvular heart disease. Research interests include fusion imaging and valve interventions, alongside teaching and mentoring graduate students.
