# Supplementary figures and images for: Artificial intelligence based fusion imaging streamlining mitral transcatheter edge-to-edge repair
Source: Eur Heart J Imaging Methods Pract. 2026 Mar 12;4(1):qyag048. doi: 10.1093/ehjimp/qyag048 (PMC13015911; doi:10.1093/ehjimp/qyag048)

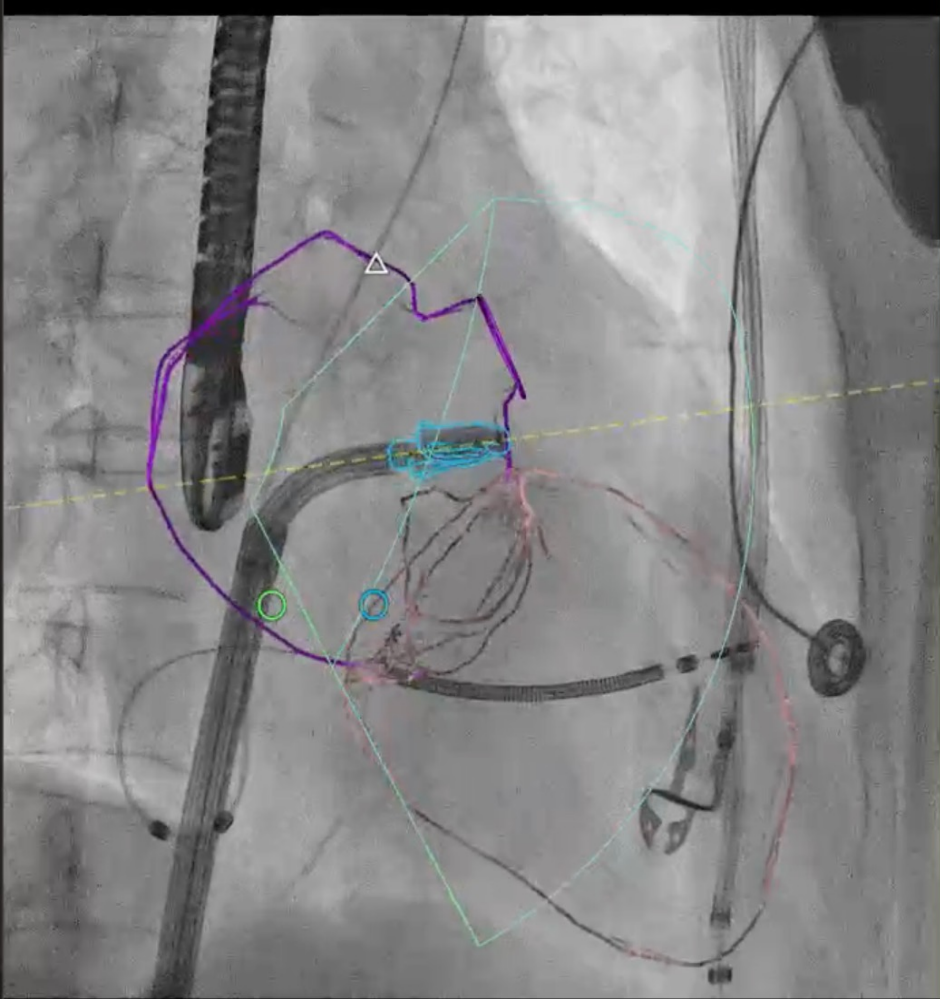

# Video 1 still image

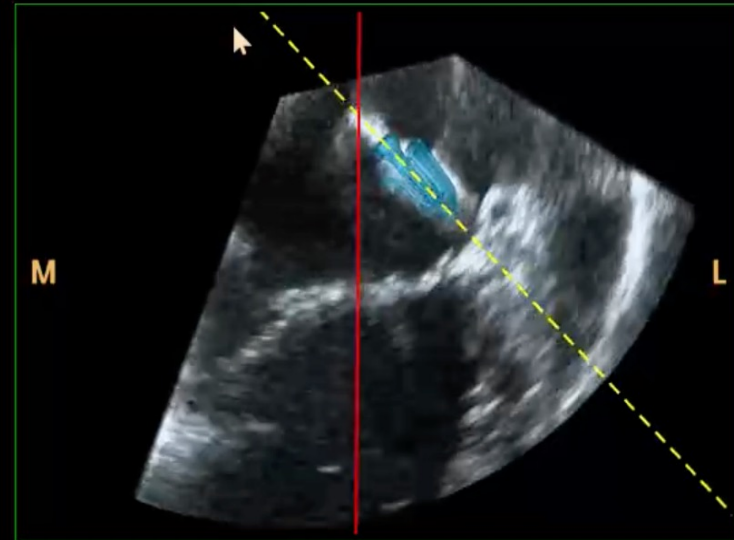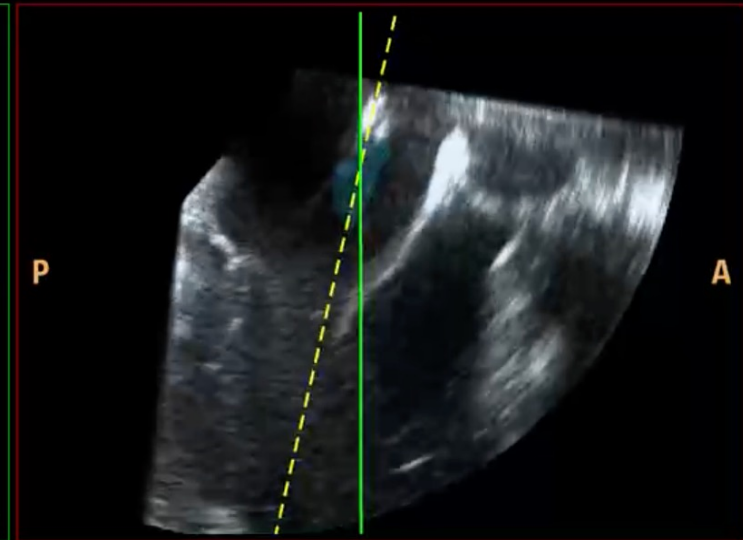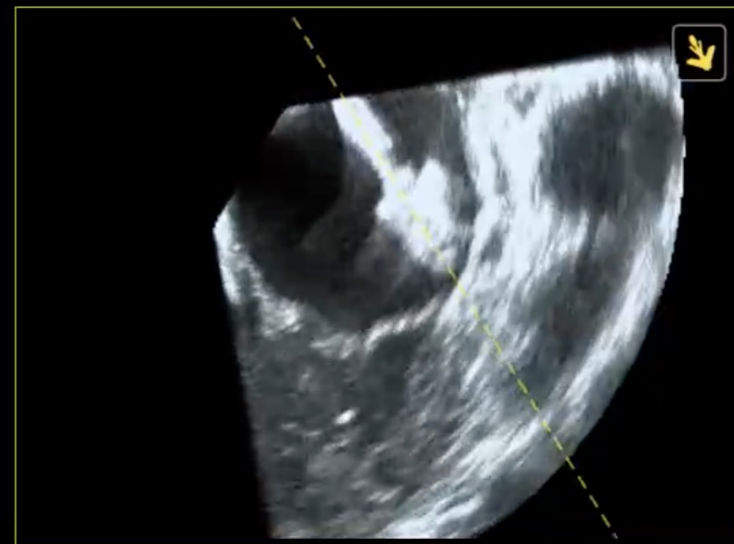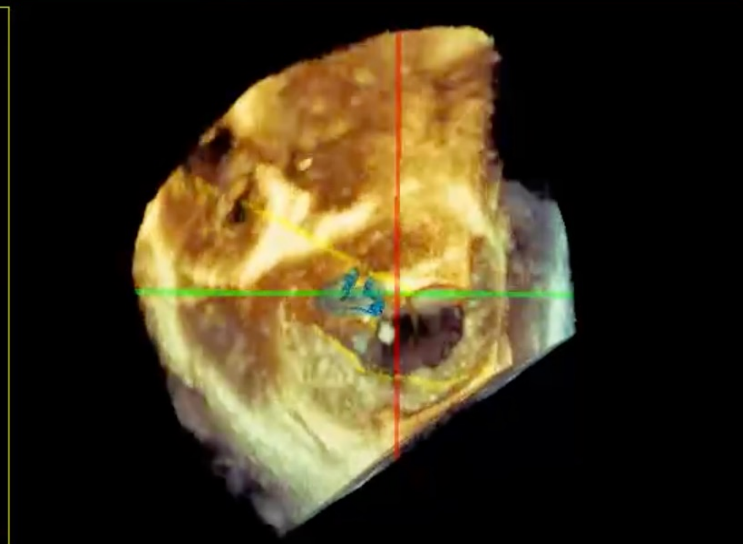

Supplement: qyag048_Supplementary_Data [file qyag048_supplementary_data.zip › video 1 still image.pdf]

X-ray 1

Echo

## Video 2 still image

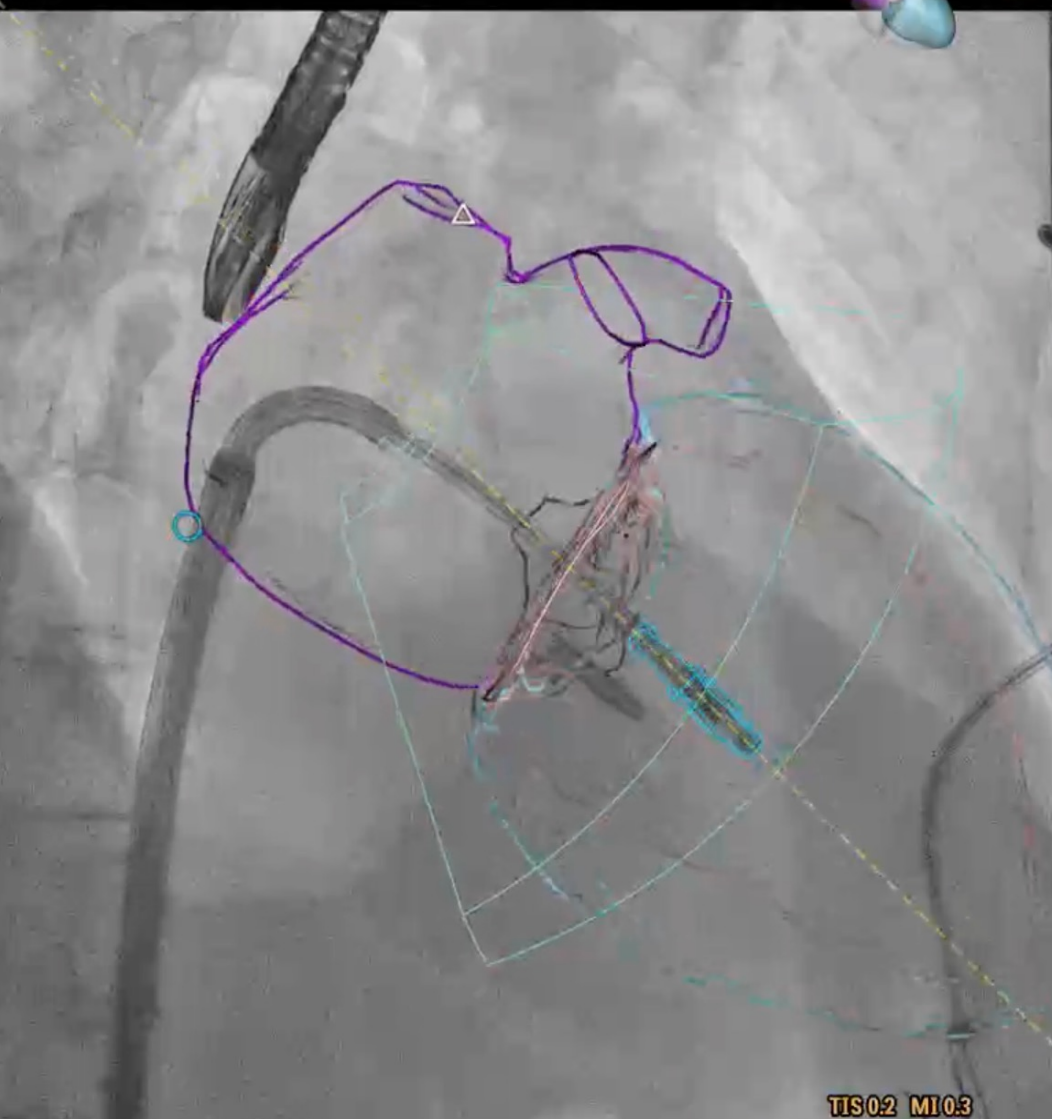

TIS02 MI0.3

RAO 36°  
CRAN 4°

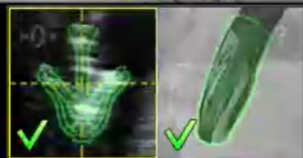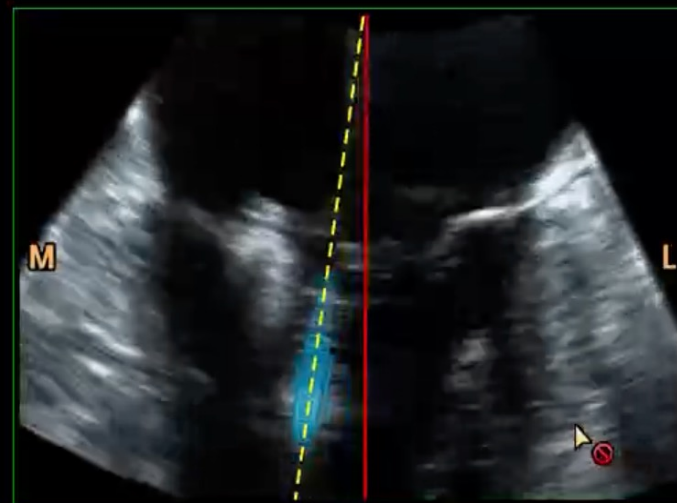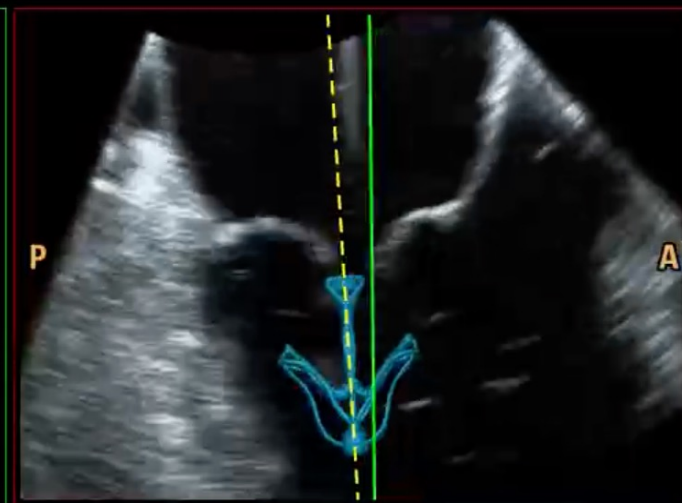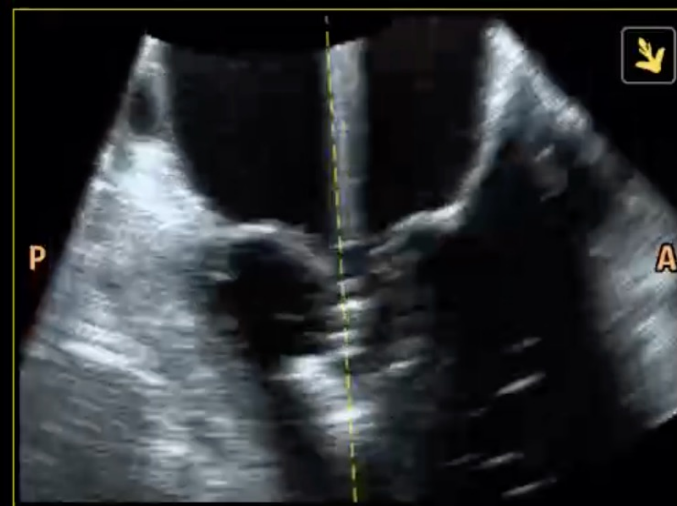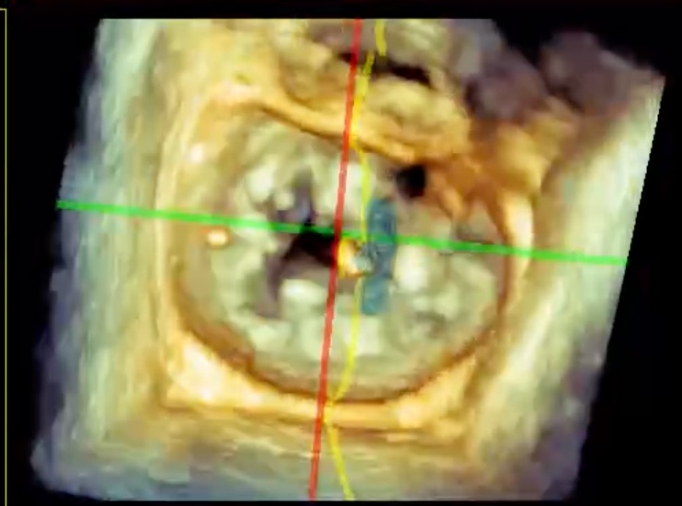

Supplement: qyag048_Supplementary_Data [file qyag048_supplementary_data.zip › video 2 still image.pdf]

# Video 3 still image

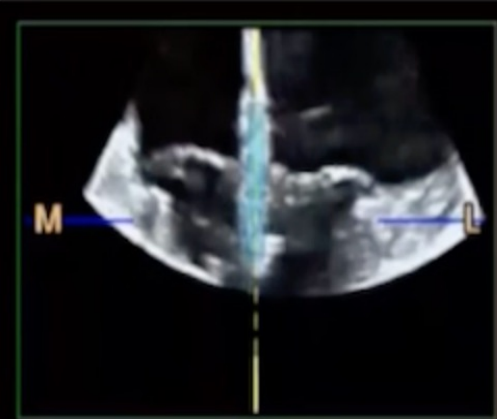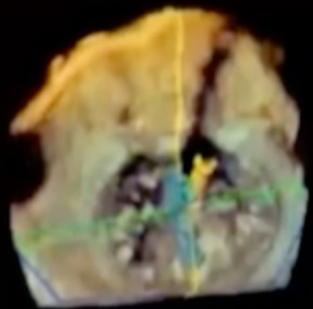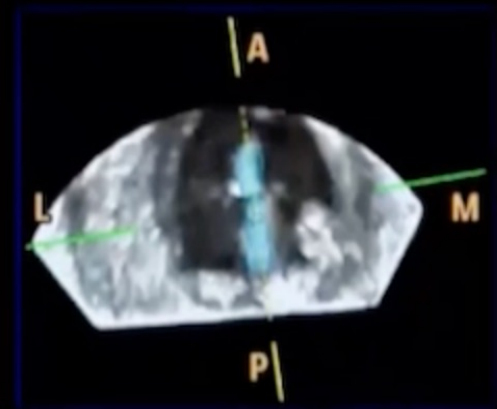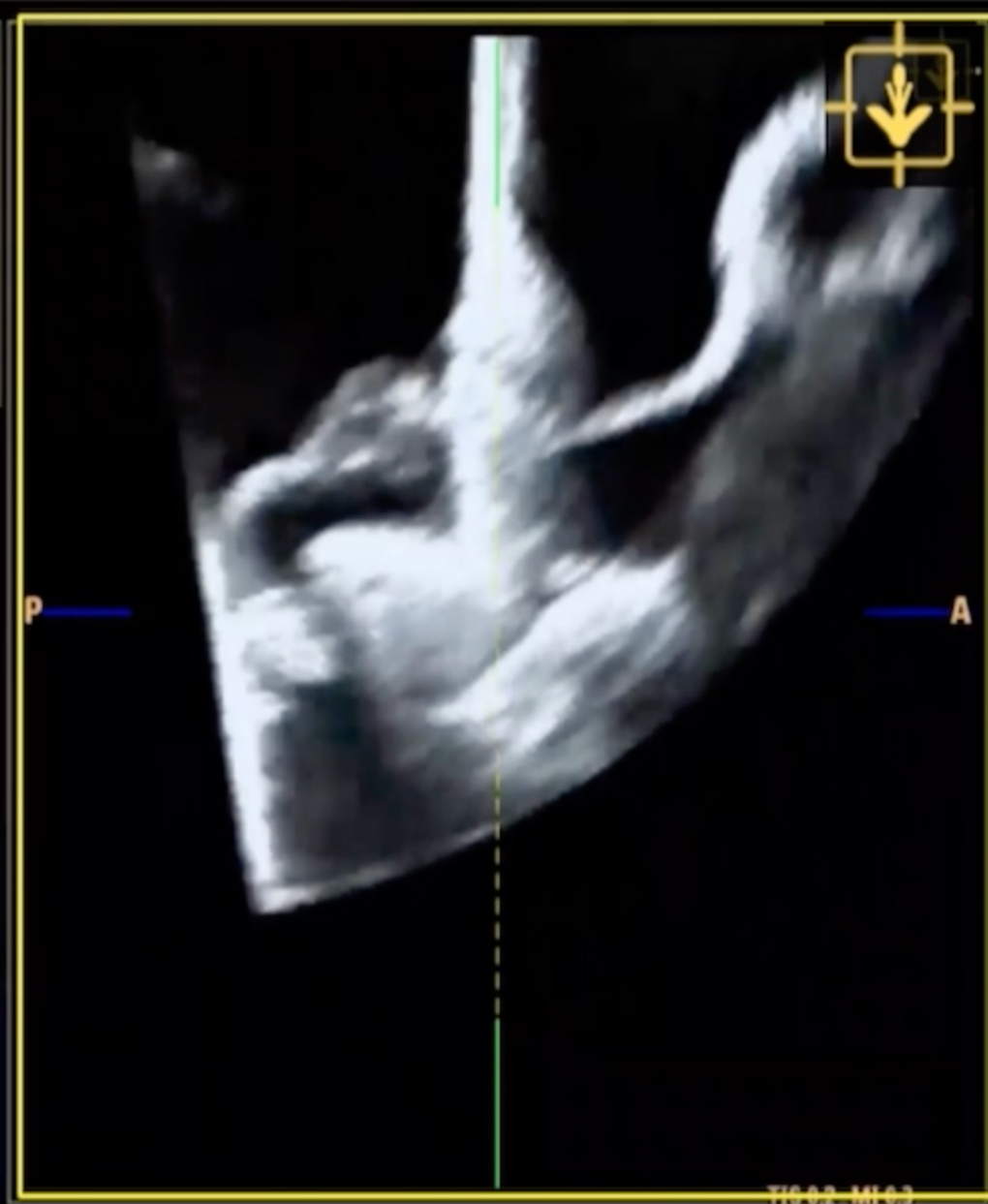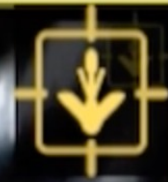

TIG 0.2 MI 0.3

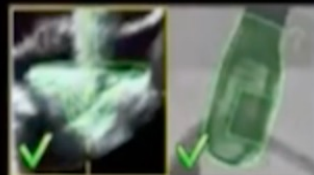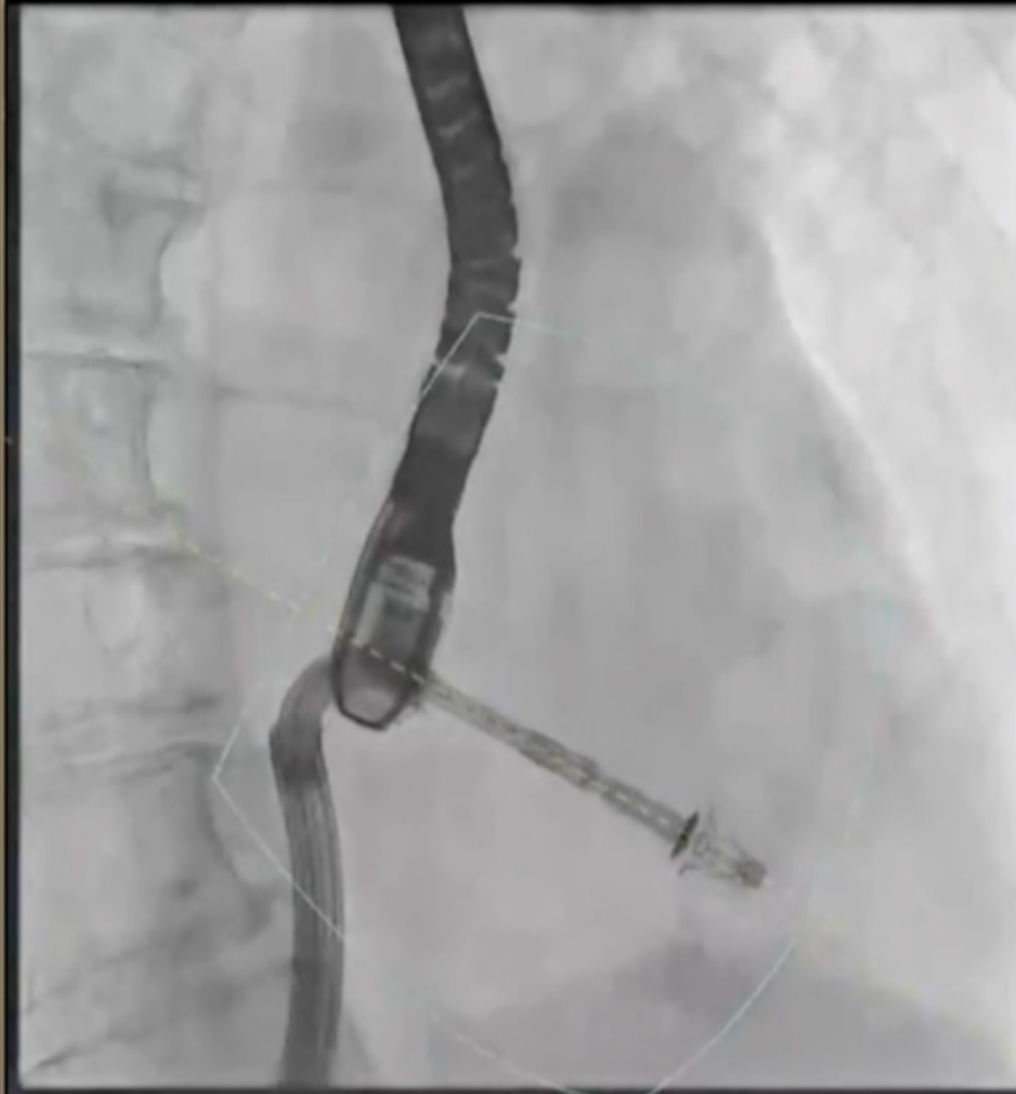

LAO 4°  
CRAN 1°

Supplement: qyag048_Supplementary_Data [file qyag048_supplementary_data.zip › video 3 still image.pdf]

# Video 4 still image

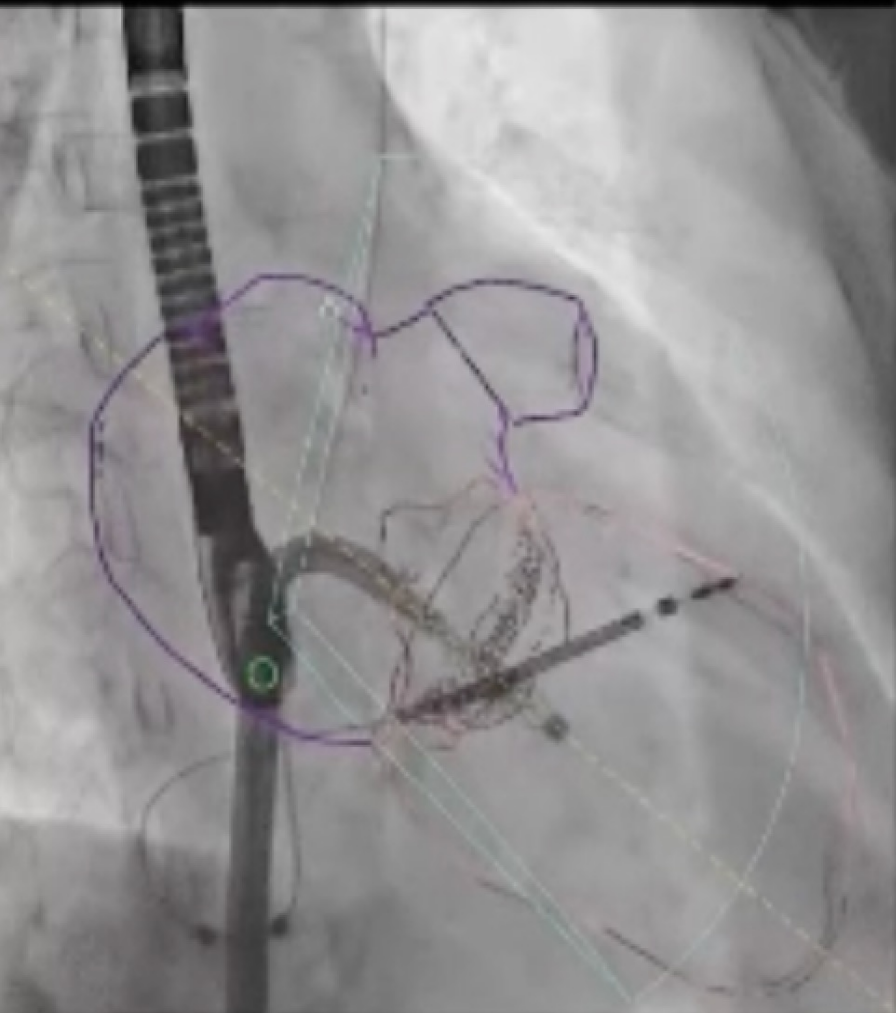

T10 L2 M142

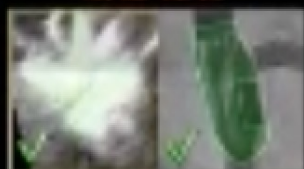

PAD 12'  
LAD 18'

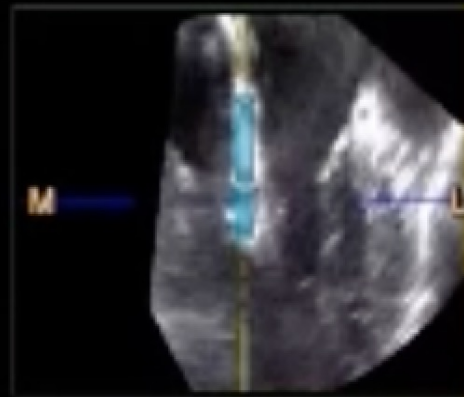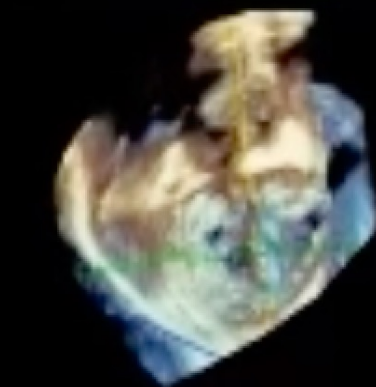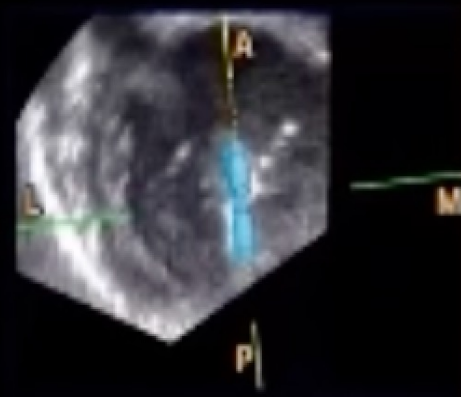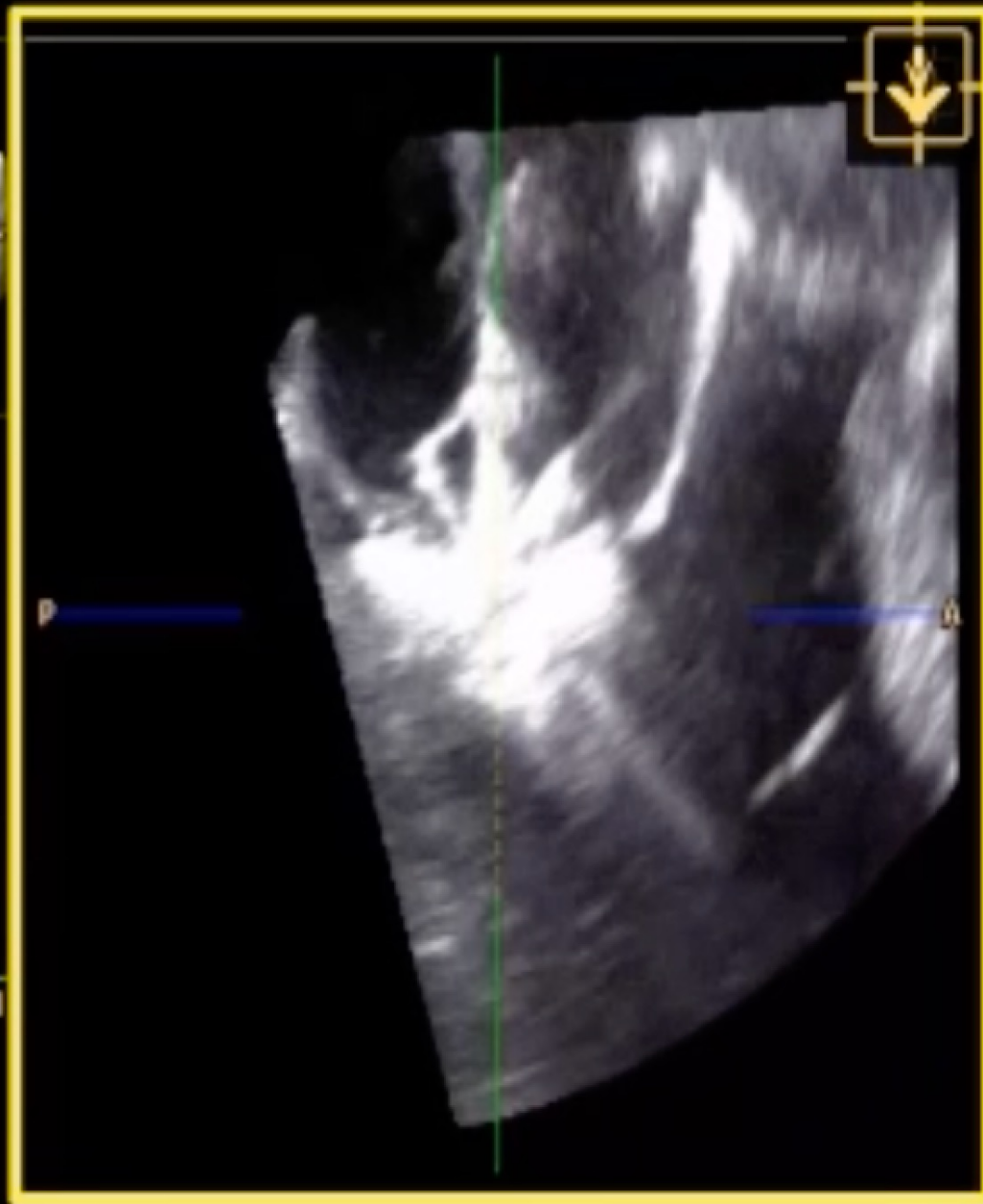

Supplement: qyag048_Supplementary_Data [file qyag048_supplementary_data.zip › video 4 still image.pdf]
